# The Role of Melatonin in Smoking Cessation and COPD Management: A Randomized Controlled Trial

**DOI:** 10.1155/carj/7322988

**Published:** 2026-09-22

**Authors:** Mohsen Gholinataj Jelodar, Negin Haddad, Farahnaz Hoseinzadeh, Samaneh Mirzaei, Adeleh Sahebnasagh, Vahid Ramezani, Mohammad Hossein Mohammadi, Fatemeh Saghafi

**Affiliations:** ^1^ Division of Pulmonary and Critical Care Medicine, Department of Internal Medicine, School of Medicine, Shahid Sadoughi University of Medical Sciences, Yazd, Iran, ssu.ac.ir; ^2^ Clinical Research Development Center, Shahid Rahnemoon Hospital, School of Medicine, ShahidSadoughi University of Medical Sciences, Yazd, Iran; ^3^ Pharmaceutical Sciences Research Center, School of Pharmacy, Shahid Sadoughi University of Medical Sciences, Yazd, Iran, ssu.ac.ir; ^4^ Department of Health in Disaster and Emergencies, School of Public Health, Shahid Sadoughi University of Medical Sciences and Health Services, Yazd, Iran, ssu.ac.ir; ^5^ Student Research Committee, Shahid Sadoughi University of Medical Sciences, Yazd, Iran, ssu.ac.ir; ^6^ Department of Internal Medicine, Clinical Research Center, Faculty of Medicine, North Khorasan University of Medical Sciences, Bojnurd, Iran, nkums.ac.ir; ^7^ Department of Pharmaceutics, School of Pharmacy, Shahid Sadoughi University of Medical Sciences, Yazd, Iran, ssu.ac.ir; ^8^ Department of Neurosurgery, School of Medicine, Shahid Sadoughi University of Medical Sciences, Yazd, Iran, ssu.ac.ir; ^9^ Traditional Pharmacy and Pharmaceutical Sciences Research Center, Department of Clinical Pharmacy, Faculty of Pharmacy, Shahid Sadoughi University of Medical Sciences, Yazd, Iran, ssu.ac.ir

**Keywords:** COPD, melatonin therapy, nicotine dependence, smoking cessation

## Abstract

**Background:**

Chronic obstructive pulmonary disease (COPD) is a leading global health concern, characterized by persistent airflow obstruction and a significant burden on quality of life. Smoking cessation is critical for COPD management, but nicotine addiction poses challenges. Emerging evidence suggests that melatonin may aid in addiction management, though its efficacy in COPD remains underexplored.

**Methods:**

This randomized, triple‐blind, controlled clinical trial evaluated the impact of melatonin on nicotine dependence and COPD outcomes over 180 days. Sixty male COPD patients with a history of smoking were randomized to receive melatonin (5 mg or placebo). Smoking behavior, psychological well‐being, and hospitalization rates were assessed using validated scales.

**Results:**

Both groups exhibited reduced cigarette consumption and improved psychological scores over time, with no significant differences between groups. Hospitalization rates due to COPD exacerbations were lower in the melatonin group (4% vs. 24% in placebo), indicating a nonsignificant trend that warrants further investigation, although the difference was not statistically significant.

**Conclusion:**

While melatonin did not significantly affect smoking cessation or psychological outcomes, the observed reduction in hospitalization rates should be interpreted cautiously as an exploratory finding. Larger, long‐term, and adequately powered studies are needed to confirm these results and further investigate the potential role of melatonin as an adjunct therapy in COPD management.

**Trial Registration:** Iranian Registry of Clinical Trials (IRCT): IRCT20190810044500N25

## 1. Introduction

Chronic obstructive pulmonary disease (COPD) is a progressive respiratory condition marked by persistent airflow obstruction, which significantly contributes to increased morbidity and mortality [1]. COPD substantially impairs quality of life, leading to disability and limiting daily activities as the disease progresses [2–4]. COPD is the third leading cause of death globally and is associated with a rising prevalence, making it a major concern for healthcare professionals. The World Health Organization (WHO) estimates that around 80 million people suffer from moderate to severe COPD, resulting in three million deaths in 2005, highlighting its substantial public health impact [5].

Effective COPD management should be personalized to address disease heterogeneity, severity, and comorbidities, emphasizing the need for ongoing research to optimize treatment strategies [6]. In addition to pharmacological treatments, nonpharmacological strategies are commonly employed to manage COPD patients [7, 8]. Nonpharmacological strategies encompass pulmonary rehabilitation to enhance physical conditioning, smoking cessation to slow disease progression, and patient education to improve adherence to treatment [8, 9].

Smoking is the primary cause of COPD. It contributes to chronic inflammation and airway obstruction [10]. This underscores the need for comprehensive strategies to address tobacco use in COPD prevention and management. Continued tobacco use is projected to result in millions of deaths and substantial healthcare costs, with approximately 90% of COPD‐related mortality attributed to cigarette smoking. Studies indicate that early smoking cessation can significantly slow the progression of this debilitating respiratory condition [11]. Patients with COPD require greater support than smokers without comorbidities, especially given that a substantial proportion of COPD patients are smokers. Consequently, prioritizing smoking cessation interventions is critical. These interventions are cost‐effective, significantly attenuate the decline in lung function, and reduce complications and mortality associated with the disease [2, 12]. According to the traditional view, nicotine in cigarettes is a highly addictive substance, which makes quitting smoking difficult, especially for heavy smokers [13]. Nicotine intake produces a subjectively pleasant experience (reward), the obtaining of which increases the likelihood that smoking behavior will occur again (reinforcement) [14].

Melatonin, a neurohormone synthesized in the pineal gland, is crucial in regulating circadian rhythms, reproductive functions, neurobehavior, antioxidant defenses, and immune responses [15]. Its biological effects are mediated through melatonin receptors MT1 and MT2, with the MT1 receptor localized in key brain regions such as the prefrontal cortex, hippocampus, nucleus accumbens, and amygdala, all of which are implicated in addiction‐related behaviors. Current research indicates that core genes involved in circadian regulation also modulate reward‐related behaviors associated with substance use disorders. Additionally, the presence of melatonin within the dopaminergic system suggests its potential involvement in the neurobiology of drug addiction. Preclinical studies have demonstrated that exogenous melatonin supplementation, along with the manipulation of its receptor pathways, may effectively mitigate addiction‐related behaviors. Notably, melatonin exhibits an inverse relationship with dopamine in specific tissues. Investigations utilizing a pheochromocytoma cell line have shown that melatonin can dose‐dependently inhibit nicotine‐induced dopamine release. These findings highlight melatonin’s therapeutic potential for central nervous system disorders, including substance use disorders [15–18].

To date, various trials have examined the effectiveness of melatonin on addiction to various substances, including benzodiazepines, alcohol, opioids, and nicotine. The results of previous studies on the use of exogenous melatonin in substance addiction have been inconsistent, and we currently do not have sufficient robust evidence to support the use of this treatment in patients. According to our best available research, in the only randomized clinical trial conducted on the impact of melatonin on nicotine withdrawal, Zhdanova and Piotrovskaya administered a single oral dose of 0.3 mg of melatonin to regular smokers after 3.5 h of nicotine withdrawal. They found that despite a reduction in self‐reported mood ratings using a visual analog scale (VAS), there was no significant effect on performance tests. Based on the results of the study, melatonin may help reduce mood changes associated with nicotine withdrawal. The use of a single dose of low‐dose melatonin may have contributed to the results [19].

In light of the limited research investigating the effects of melatonin on smoking cessation, especially in patients with COPD, there is a compelling need for studies employing this agent in smokers. Such investigations are essential to comprehensively evaluate the efficacy of melatonin in mitigating nicotine addiction. This study was proposed to address this significant gap in the current scientific literature.

## 2. Materials and Methods

The study adhered to the ethical standards of the Declaration of Helsinki (1975, revised 2013) [20], with all treatments provided at no additional cost beyond standard expenses. Inclusion criteria included age ≤ 75 years, confirmed COPD Stage I or II [21] for at least 12 months, smoking ≥ 10 cigarettes/day over the past year, willingness to quit, and full commitment to study participation. A pulmonologist confirmed the COPD diagnosis and staging. The diagnosis of airflow limitation and its severity was according to criteria of global initiative for chronic obstructive lung disease (GOLD) and Spirometry indices in the recent year. The FEV1 to FVC ratio of less than 0.7 was required to confirm airway obstruction.

Exclusion criteria included significant mental health disorders (e.g., major depressive disorder, panic disorder, or bipolar disorder), a recent history of alcohol or substance use disorder (within 12 months), concurrent use of medications that could interfere with the study drugs (such as nicotine replacement therapy), a body mass index (BMI) below 18.5 or above 34.9, chronic medical conditions that could hinder consistent follow‐up, and pregnancy, breastfeeding, or plans to conceive during the study.

### 2.1. Intervention, Randomization, and Blinding

Once eligibility criteria were met, patients were randomly assigned, according to a random number table, to receive either a 5‐mg melatonin tablet or a placebo. Blinding was applied to participants, the clinical response evaluator, and the pulmonologist to ensure an unbiased outcome assessment and was maintained throughout both the intervention and follow‐up periods until completion of statistical analysis. Placebo tablets were identical to melatonin tablets in appearance, packaging, and administration schedule. A formal blinding assessment was conducted at the end of the study by asking participants and outcome assessors to guess their assigned group.

Patients were instructed to take one tablet nightly before bed and avoid other medications or supplements during the study. They were also advised to avoid using other medications or supplements during the treatment period. The duration of drug use in both study groups was 180 ± 7 days. After 180 days of intervention, participants were followed up for an additional 2 months, totaling 8 months of observation, with a final follow‐up visit. The placebo was prepared in the laboratory of the Faculty of Pharmacy, Yazd University of Medical Sciences, with a similar appearance and shape to the melatonin tablet. Both drugs were given to the patients in a completely similar shape at 1‐month intervals. In order to follow up the patients for medication adherence, patients were asked to return the empty bottle at the end of each month.

### 2.2. Data Collection and Outcome Measures

Participants were followed up for 180 days of intervention plus an additional 2 months, totaling 8 months, with a final follow‐up visit. At enrollment, baseline demographic and clinical characteristics were collected, as outlined in Table 1, including age, BMI, lung function parameters (FEV1 and FEV1/FVC), smoking history (pack/year consumption), and the presence of underlying diseases or concurrent medication use.

**TABLE 1:** Demographic and clinical features of patients.

Characteristic	Groups		*p*
	Melatonin *N* = 25	Placebo *N* = 25	
Age, Mean (SD)	60.4 (7.3)	60.3 (8.5)	0.944
BMI, Mean (SD)	23.2 (2.6)	22.8 (3.2)	0.582
FEV1, Mean (SD)	71.0 (6.5)	69.1 (8.3)	0.376
FEV1/FVC, Mean (SD)	63.4 (3.5)	62.2 (4.7)	0.321
Smoking Habit, Median (IQR)			
Pack/year	30.0 (29.0)	40.0 (32.5)	0.296
Underlying disease, N (%)			
CVD	0 (0.0)	1 (4.0)	0.175
HTN	2 (8.0)	4 (12.0)	
DM	1 (4.0)	3 (12.0)	
RA	1 (4.0)	0 (0.0)	
DLP	0 (0)	1 (4)	
Concurrent medications, N (%)			
*β*‐blockers	1 (4.0)	2 (8.0)	0.297
NSAIDs	1 (4.0)	0 (0.0)	
Biguanides	0 (0.0)	3 (12.0)	
ACEI/ARBs	0 (0.0)	3 (12.0)	
Statins	0 (0.0)	1 (4.0)	
Others	2 (8.0)	5 (20.0)	

*Note:* DLP, dyslipidemia; HTN, hypertension; IQR, interquartile range; RA, rheumatoid arthritis.

Abbreviations: ACEI, angiotensin‐converting enzyme inhibitor; ARB, angiotensin receptor blockers; BMI, body mass index; CVD, cardiovascular diseases; DM, diabetes mellitus; FEV1, forced expiratory volume in 1 s, FVC, forced vital capacity; N, number; NSAIDs, nonsteroid anti‐inflammatory drugs; SD, standard deviation.

To ensure adherence to the protocol, patients received a 1‐month supply of either melatonin or placebo tablets at each visit, along with detailed instructions on proper usage. Scheduled follow‐up assessments were conducted on days 1, 90 ± 7, and 180 ± 7 of the intervention period. After completion of the 180‐day intervention, participants were followed up for an additional 2 months. This final follow‐up visit corresponded to approximately Month 8 of the study timeline. During all scheduled visits, cigarette consumption and psychological measures were systematically collected, and hospitalization outcomes related to COPD exacerbations were continuously monitored throughout the study period. Daily cigarette consumption was recorded at each visit based on participant self‐report, and individual‐level patterns of reduction were examined to distinguish between gradual decreases across most participants versus complete cessation in a minority. The desire to quit smoking in patients was assessed through the VAS [22]: A measure of self‐efficacy and motivation, ranging from 0 (low) to 10 [23].

The following questionnaires were used to collect data related to mood disorders, nicotine dependence, and smoking behaviors in the participants.

Fagerström Test for Nicotine Dependence (FTND) [24]: A six‐item measure categorizing nicotine dependence into mild (0–3 points), moderate (4–6 points), and severe (7–10 points) levels.

Beck Depression Inventory‐II (BDI‐II) [25]: A 21‐item self‐assessment for depressive symptoms, with scores of 0–9 indicating minimal depression, 10–18 indicating mild to moderate depression, and 19–29 indicating moderate to severe depression.

Beck Anxiety Inventory (BAI) [26]: A 21‐item self‐assessment for anxiety, with scores of 0–7 representing minimal anxiety, 8–15 mild anxiety, 16–25 moderate anxiety, and 26–63 severe anxiety.

Glover–Nilsson Smoking Behavioral Questionnaire (GN‐SBQ) [27]: An assessment of smoking‐related behaviors and thoughts, with scores below 12 indicating mild dependence, 12–22 moderate dependence, 23–33 strong dependence, and above 33 extreme dependence.

The Pittsburgh Sleep Quality Index (PSQI): A self‐report questionnaire to assess sleep quality. It evaluates seven components: subjective sleep quality, sleep latency, sleep duration, habitual sleep efficiency, sleep disturbances, use of sleep medication, and daytime dysfunction. Each component is scored from 0 to 3, with higher scores indicating poorer sleep quality. The sum of these component scores yields a global score ranging from 0 to 21, where a higher score reflects worse sleep quality [28].

### 2.3. Sample Size Calculation

Considering a significance level of 5% and a power of 80%, and based on a similar study [19], the sample size for each group was calculated to be 25 participants to detect a statistically significant difference of at least 2 units in the mean VAS score with a standard deviation of 2.5. To account for a potential 10% dropout rate, the target sample size was increased to 28 participants per group. For added reliability, 30 participants were enrolled in each group.

### 2.4. Statistical Analysis

The study data were collected, entered into a database, and analyzed using SPSS software, version 20. Descriptive statistics, including frequencies, percentages, means, and standard deviations, were presented in the form of tables and charts. For data analysis, a range of statistical tests were employed: the chi‐squared test for comparing categorical data between two groups, the independent‐samples *T*‐test for comparing continuous data between two groups, the paired‐samples *T*‐test for comparing pre‐ and post‐treatment continuous data within groups, the Mann–Whitney U test for comparing ordinal data between groups, and repeated measures ANOVA for assessing within‐group and between‐group changes over time. A p of < 0.05 was considered statistically significant for all tests.

## 3. Results

**FIGURE 1:**
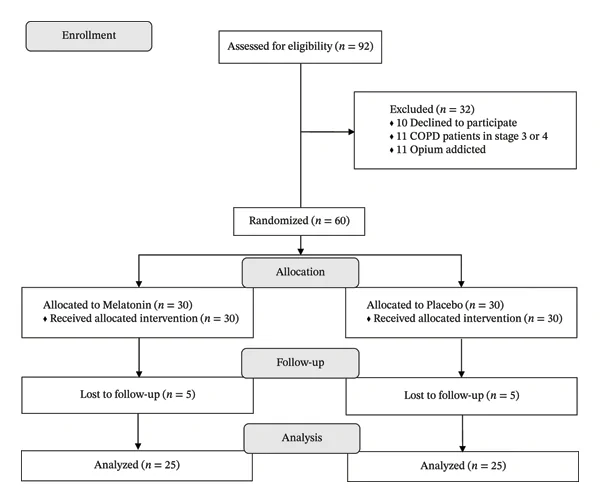
CONSORT flow diagram of melatonin vs. placebo during study follow‐up.

A total of 60 participants were assessed for eligibility, of whom 60 were successfully randomized into two groups: melatonin (*n* = 30) and placebo (*n* = 30). During the follow‐up period, a small number of participants in each group were lost to follow‐up and no secondary information was obtained from them during the first 3 months of the study. Consequently, the final analysis included data from 25 participants in each group (Figure 1).

### 3.1. Baseline Demographic and Clinical Characteristics

Table 1 summarizes the baseline characteristics of male participants enrolled in the study. The randomization process resulted in well‐matched groups, with no significant differences in key demographic or clinical features. The mean age of participants was 60.4 ± 7.3 years in the melatonin group and 60.3 ± 8.5 years in the placebo group (p = 0.944), reflecting a homogeneous age distribution. Similarly, the BMI was 23.2 ± 2.6 kg/m^2^ in the melatonin group and 22.8 ± 3.2 kg/m^2^ in the placebo group (p = 0.582).

Lung function metrics, including forced expiratory volume in 1 s (FEV1) and the FEV1 to FVC (forced vital capacity) ratio, were comparable between groups (*p* = 0.376 and *p* = 0.321, respectively), suggesting equivalent baseline pulmonary status. Additionally, there was no significant difference between groups in terms of smoking habits. The prevalence of comorbidities, such as hypertension (HTN), diabetes mellitus (DM), and dyslipidemia (DLP), was low and balanced between the two groups. For instance, DM was present in 4% of the melatonin group versus 12% in the placebo group. The groups also showed no significant differences in concurrent medication use, including *β*‐blockers, NSAIDs, ACE inhibitors/ARBs, and biguanides.

### 3.2. Primary and Secondary Outcomes Over 180 Days

Table 2 presents the longitudinal assessment of smoking‐related behaviors, psychological well‐being, and quality‐of‐life measures. Outcomes were evaluated at baseline, Days 90 and 180, and 2 months postintervention to track changes over time.

**TABLE 2:** Primary and secondary outcomes measured at baseline, Months 3 and 6, and at the 8‐month follow‐up.

Variables^1^	Month	Melatonin *N* = 25	Placebo *N* = 25	Between groups *p*	Effect of time *p*	Effect of time × group *p*	Overall *p*
Cigarettes	0	20.0 (8.0)	20.0 (10.0)	0.765	**<** **0.001** ^2^	0.115	0.384
	3	20.0 (10.0)	20.0 (13.0)	0.968			
	6	15.0 (15.0)	20.0 (10.0)	0.519			
	8	20.0 (15.0)	20.0 (7.0)	0.188			

Fagerstrom	0	5.0 (2.0)	5.0 (1.0)	0.681	**<** **0.001**	0.155	0.401
	3	4.0 (3.0)	4.0 (4.0)	0.992			
	6	4.0 (3.0)	4.0 (3.0)	0.473			
	8	4.0 (3.0)	5.0 (1.0)	0.198			

GN	0	13.0 (4.0)	13.0 (8.0)	0.792	**<** **0.001**	0.376	0.767
	3	12.0 (7.0)	11.0 (7.0)	0.606			
	6	9.0 (8.0)	10.0 (6.0)	0.654			
	8	9.0 (8.0)	10.0 (8.0)	0.726			

BDI	0	4.0 (3.0)	4.0 (2.0)	0.890	**<** **0.001**	0.159	0.978
	3	4.0 (2.0)	4.0 (1.0)	0.374			
	6	4.0 (2.0)	5.0 (3.0)	0.505			
	8	5.0 (3.0)	5.0 (1.0)	0.653			

BAI	0	4.0 (3.0)	4.0 (4.0)	0.844	**0.012**	0.218	0.242
	3	4.0 (2.0)	4.0 (5.0)	0.441			
	6	4.0 (2.0)	4.0 (4.0)	0.241			
	8	4.0 (2.0)	5.0 (4.0)	0.342			

VAS	0	6.0 (2.0)	6.0 (2.3)	0.708	**<** **0.001**	0.445	0.335
	3	5.0 (2.0)	4.5 (2.0)	0.715			
	6	4.0 (2.3)	5.0 (2.0)	0.236			
	8	5.0 (1.0)	5.0 (1.0)	0.134			

PSQI	0	8.0 (3.0)	7.0 (3.0)	0.312	**<** **0.001**	**<** **0.001**	0.083
	3	6.0 (4.0)	7.0 (2.0)	0.031			
	6	5.0 (3.0)	8.0 (3.0)	< 0.001			
	8	7.0 (3.0)	7.0 (2.0)	0.890			

^1^Data based on median (interquartile range).

^2^Values below 0.001 reported as < 0.001 to ensure consistency and clarity.

Abbreviations: BAI, Beck’s Anxiety Inventory; BDI, Beck’s Depression Inventory; GN, Glover–Nilsson; PSQI, Pittsburgh Sleep Quality Index; VAS, Visual Analogue Scale.

### 3.3. Smoking Behavior

The number of cigarettes smoked per day exhibited a significant effect of time, while no significant group effect (*p* = 0.384) or time × group interaction was observed. Cigarette consumption decreased from baseline to follow‐up in both groups over time, consistent with a temporal effect rather than a treatment‐specific effect. Similarly, scores on the Fagerström Test for Nicotine Dependence showed a marginal decline over time, with no significant differences between groups (*p* = 0.401) and no significant group or time × group interaction effects.

### 3.4. Psychological and Quality‐of‐Life Outcomes

The study also evaluated psychological outcomes using the BDI and BAI and smoking behavioral dependence using the GN‐SBQ scale.

BDI: Depression scores were stable throughout the study, with no significant differences between the groups at any time point (*p* = 0.978) and no significant time × group interaction.

BAI: Anxiety levels showed slight improvement over time in both groups, although the between‐group comparisons were nonsignificant (*p* = 0.242) with no significant time × group interaction observed.

GN‐SBQ and VAS: Behavioral dependence and quality‐of‐life scores showed modest improvements. By Day 180, the median GN‐SBQ score was 9.0 in the melatonin group and 10.0 in the placebo group (*p* = 0.767). VAS scores improved slightly in both groups (*p* = 0.335), with no significant group or time × group interaction effects.

PSQI: The results showed significant improvement in the melatonin group over time. At baseline, PSQI scores were similar between groups (*p* = 0.312). By the third month, sleep quality significantly improved in the melatonin group (*p* = 0.031) and continued to do so at the sixth month (*p* < 0.001). However, by the follow‐up period, scores in both groups returned to similar levels (*p* = 0.890), suggesting a primarily time‐dependent pattern rather than a sustained treatment effect of melatonin.

### 3.5. Hospitalization Outcomes

Hospitalization due to COPD exacerbations occurred less frequently in the melatonin group (4%; 1/25) compared with that in the placebo group (24%; 6/25). Although this difference did not reach statistical significance (*p* = 0.098), it may indicate a potential clinical difference; however, no formal time ×  group analysis was performed for this secondary outcome. Given the relatively small sample size, the exploratory nature of this outcome, and the small number of events, these findings should be interpreted cautiously.

The absolute risk of hospitalization due to COPD exacerbation was 24% in the placebo group compared with 4% in the melatonin group, corresponding to a relative risk of 0.17 (95% CI: 0.02–1.30) and an absolute risk reduction of 20%. The number needed to treat (NNT) was 5; however, these estimates should be interpreted cautiously because the confidence interval was wide and the between‐group difference was not statistically significant.

Hospitalizations unrelated to exacerbations were infrequent and identical between groups, occurring in 4% of participants (1/25) in each group (*p* = 1.000).

## 4. Discussion

This randomized clinical trial examined the effects of melatonin compared to placebo on smoking behaviors, psychological well‐being, and sleep quality of COPD patients over an 8‐month period. Both groups demonstrated improvements over time; however, no significant between‐group differences were observed across primary and secondary smoking‐related outcomes. Overall, melatonin at the tested dose and duration without concurrent behavioral therapy did not demonstrate clinically meaningful efficacy in smoking cessation, nicotine dependence, or psychological well‐being. These findings should be interpreted cautiously given the limited sample size and imprecision of the effect estimates.

Interactions between melatonin and nicotine dependence have been identified in preclinical studies, suggesting that nicotine consumption is influenced by both exogenous and endogenous melatonin via MT1/MT2 receptors [29]. In human studies, low‐dose melatonin administered during acute nicotine withdrawal reduced self‐reported mood disorders and craving but did not significantly affect functional outcomes [19]. Although numerical improvements occurred in both groups, these differences were not statistically significant and should not be interpreted as therapeutic effect. Given the limited sample size and wide uncertainty around the estimates, small between‐group differences cannot be definitively excluded. Mechanistically, melatonin may influence nicotine dependence through MT1/MT2 receptor‐mediated modulation of the mesolimbic dopamine reward pathway, which plays a central role in nicotine reinforcement and craving. Preclinical evidence suggests that melatonin can attenuate dopamine release in reward‐related brain regions, thereby providing a biological rationale for its potential role in addiction modulation, even though these effects were not translated into significant behavioral changes in the present clinical trial.

The observed reduction in the average daily cigarette consumption was primarily the result of a moderate decrease among most participants, rather than being driven by a small subset of patients who quit smoking completely. This suggests a uniform temporal reduction rather than a treatment‐specific effect. Consistent with this observation, melatonin did not lead to significant reduction in cigarette consumption or nicotine dependence scores (FTND). The absence of structured behavioral support in this trial may have contributed to this limited effect, in line with prior evidence suggesting that pharmacological monotherapies often show modest efficacy when used alone [30–32]. Smoking cessation is a complex behavioral and physiological process shaped by psychological support, pharmacological aids, and individual motivation. Accordingly, behavioral interventions represent a central component of smoking cessation programs, as they address learned smoking behaviors, contextual triggers, and environmental factors that make quitting difficult [33].

These methods help patients adopt alternative behaviors, including new coping strategies, healthy activities, and relaxation techniques [34]. Intensive interventions, such as individual or group counseling, further enhance cessation success. However, it should also be noted that these interventions result in greater material and time costs for program implementation and effectiveness [34]. The importance of nonpharmacological smoking cessation, even alone and without pharmacological interventions, has been demonstrated. Behavioral interventions alone, such as in‐person behavioral support and counseling, telephone counseling, and self‐help materials, can lead to increased smoking cessation success [35]. The absence of structured behavioral support in this study may have attenuated the ability to detect a potential adjunctive effect of melatonin.

In the present clinical trial, the role of melatonin in modulating psychological well‐being was examined. Both groups exhibited mild improvements in anxiety and depression scores over time, as measured by the BAI and BDI. However, these changes were comparable between the melatonin and placebo groups, indicating no additional psychological benefit from melatonin, likely due to low baseline symptom severity, which limited the potential for detectable improvement. This finding is in contrast to earlier studies, which demonstrated that melatonin’s anxiolytic and antidepressant effects may alleviate withdrawal‐related psychological distress [16, 36, 37]. These findings should therefore be considered inconclusive rather than indicative of the absence of effect. It should also be noted that improvements in sleep quality (PSQI) may have partially contributed to the observed changes in psychological outcomes (BDI and BAI), as improved sleep is independently associated with better mood and reduced anxiety.

The absence of significant changes in smoking behavior outcomes emphasizes the importance of integrating pharmacological interventions like melatonin with established behavioral and nicotine replacement therapies. A combination of pharmacotherapy with structured behavioral therapy is more effective than either approach alone [38–40]. This study focused solely on pharmacological intervention, and the absence of concurrent structured behavioral therapy represents a limitation. Future studies should explore whether adding structured behavioral interventions enhances the potential benefits of melatonin. Our findings should not be interpreted as evidence against melatonin use in combination therapy settings.

Tashkin et al. [41] highlighted the efficacy and safety of varenicline in smoking cessation for patients with mild to moderate COPD, demonstrating a significantly higher continuous abstinence rate (CAR) compared to placebo. The results showed that varenicline led to a higher CAR both in the short term (weeks 9–12) and long term (weeks 9–52), underscoring its potential as an effective intervention for smoking cessation in this population. While the treatment was associated with common side effects such as nausea, abnormal dreams, upper respiratory tract infection, and insomnia, serious adverse events were rare. Notably, two deaths occurred in the varenicline group, and one in the placebo group, although psychiatric adverse events were similar between the two groups. These findings support the use of varenicline in COPD patients while also highlighting the importance of monitoring for potential side effects. In another study [42], it was demonstrated that sustained‐release (SR) bupropion is an effective and well‐tolerated aid for smoking cessation in patients with mild to moderate COPD. The results showed significantly higher CARs from smoking in the bupropion SR group compared to those in the placebo group, both in the short term (weeks 4–7) and the longer term (weeks 4–12 and 4–26). Additionally, SR bupropion attenuated symptoms of tobacco craving and withdrawal. However, the treatment was associated with some adverse events, including insomnia (12% in the placebo group vs. 24% in the bupropion SR group). This transient improvement in sleep quality (PSQI) at Months 3 and 6 in the melatonin group suggests a potential physiologically relevant effect of melatonin on sleep, distinct from its lack of efficacy on smoking behavior or psychological outcomes. Given melatonin’s established role in circadian rhythm regulation and sleep initiation, this finding is consistent with the existing literature. These comparisons should be interpreted cautiously, as the present study was not designed for head‐to‐head efficacy evaluation.

Evidence suggests that smoking‐related behaviors have a circadian rhythm, and nicotine craving and withdrawal symptoms often show diurnal variations [43, 44]. Melatonin may play a role in the circadian regulation of nicotine craving through its effects on reward‐related pathways in the brain as well as neurotransmitter systems [45]. Given melatonin’s short half‐life and the diurnal variations in nicotine craving, the timing of administration and formulation may affect the efficacy of melatonin. Morning dosing or SR formulations of melatonin could have positive clinical effects, and future studies should explore these strategies. These hypotheses require confirmation in adequately powered trials.

Based on the results of a study in Korea of male workers who attempted to quit smoking, working night shifts was identified as a risk factor for failure to quit smoking [46]. One of the main reasons for the higher rate of smoking cessation failure in night workers compared with that in day workers can be seen in the changes in circadian rhythm disruption. The use of chronotherapy strategies including melatonin supplements and light therapy, which typically involves exposure to bright artificial light at specific times of the day, has been suggested as a promising adjunctive treatment for nicotine dependence that can help regulate sleep–wake cycles and improve mood, potentially reducing nicotine cravings and withdrawal symptoms [47]. Sleep disturbances occur in both chronic nicotine addiction and withdrawal, where melatonin may improve symptoms due to its effects on sleep regulation [16]. The improvement in sleep quality distinguishes melatonin from other treatments that may worsen insomnia. However, these considerations remain speculative in the context of the present study.

Other strengths of the study include its randomized controlled design, well‐matched baseline characteristics, and comprehensive follow‐up assessments. The study design allowed for a rigorous comparison between melatonin and placebo while controlling for confounding variables through randomization. Furthermore, the detailed collection of smoking behavior data, psychological assessments, and clinical outcomes at multiple follow‐up points added depth to the evaluation of the intervention’s impact. These strengths contribute to the reliability of the findings, particularly in terms of understanding the short‐term effects of melatonin on smoking cessation and related health outcomes. Taken together, these strengths support the internal validity of the study and the robustness of the observed findings.

Furthermore, while our results indicated a lower hospitalization rate due to COPD exacerbations in the melatonin group compared to that in placebo, it is important to emphasize that the absolute numbers were very small (one vs. six participants), and hospitalization was not the primary outcome of this study. The observed reduction in hospitalizations (*p* = 0.098) represented an exploratory trend rather than a statistically significant effect. The study was not prospectively powered to detect differences in hospitalization rates, and this outcome was exploratory. Therefore, although the observed effect estimate may be clinically relevant, the wide confidence interval and nonsignificant P value preclude any definitive conclusion regarding the effect of melatonin on COPD exacerbation–related hospitalization.

Given the exploratory nature of this analysis, these findings should be interpreted with caution and should not be considered clinically conclusive. The observed trend may represent a hypothesis‐generating observation rather than evidence of a treatment effect of melatonin on COPD exacerbations.

Several important limitations must be acknowledged. First, the reliance on self‐reported measures, although commonly used in smoking research, may introduce recall and reporting bias and reduce the accuracy of estimates related to smoking behavior and dependence. Additionally, smoking outcomes (cigarette consumption and FTND) were assessed using self‐reported measures without biochemical verification methods such as exhaled carbon monoxide or cotinine testing, due to logistical and financial constraints and the outpatient follow‐up design.

The relatively small sample size limited the precision of the effect estimates and increased uncertainty around the observed between‐group differences, particularly for key primary outcomes such as smoking cessation and exploratory outcomes such as hospitalization. Therefore, these findings should be interpreted as exploratory rather than confirmatory. Additionally, the inclusion of only male participants, the relatively short follow‐up period, the lack of concurrent behavioral therapy, and the absence of inflammatory biomarker measurements (e.g., CRP or proinflammatory cytokines), and the exclusion of patients with severe COPD (GOLD stage III–IV) further constrain the generalizability of the findings.

The intervention was associated with a transient improvement in sleep quality as measured by the PSQI, a finding that is clinically relevant, as improved sleep may contribute to better overall wellbeing and potentially support smoking cessation efforts. Although this effect was transient, it highlights the potential role of melatonin in enhancing sleep parameters and suggests that the study was capable of detecting changes in domains where melatonin is biologically active. However, despite these short‐term improvements, the overall effect sizes were small, and the trajectories of change between the groups were largely parallel, with no evidence of a significant treatment‐by‐time interaction. These patterns suggest that melatonin may not exert a meaningful independent effect on smoking behavior, dependence, or mood symptoms. Moreover, wide confidence intervals further highlight the uncertainty of the observed differences and preclude firm conclusions regarding equivalence. Accordingly, equivalence between groups cannot be established. Therefore, these findings warrant further investigation in larger and longer‐term studies.

Another limitation relates to dosing considerations. The standard nightly dose used in this study may not have been optimal, particularly given melatonin’s wide safety margin and known interindividual variability in responsiveness. The short half‐life of melatonin also raises pharmacokinetic concerns: evening‐only dosing may have resulted in insufficient daytime serum levels to influence nicotine craving or withdrawal. Alternative regimens such as higher doses, morning dosing, or slow‐release formulations were not evaluated and may produce different outcomes. These factors may have contributed to the lack of detectable effect.

Future research should address current limitations by including larger, more diverse populations, extending follow‐up periods, and testing different doses, formulations, or routes of melatonin administration. Incorporating biomarker analyses of systemic inflammation, oxidative stress, and neurochemical changes may further clarify the mechanisms underlying melatonin’s effects.

Combining melatonin with behavioral interventions, nicotine replacement therapies, or other pharmacological agents could enhance smoking cessation outcomes. Additionally, dose‐escalation or pharmacokinetic‐guided strategies may help optimize therapeutic exposure while maintaining tolerability. Alternative formulations designed to achieve sustained serum concentrations may also be of interest. These approaches should be considered in future trial design.

Although no gender restrictions were applied in the inclusion criteria, all participants were male, which is an important limitation. Due to cultural context, COPD etiology differed between genders in the study population. This substantially limits generalizability to both female and mixed‐sex populations.

Recent studies suggest that sex differences in circadian rhythm and melatonin physiology may influence treatment response. Females also show differences in endogenous melatonin secretion patterns independent of sex hormones [48]. These differences may lead to variability in treatment response and should be considered in future trial design. Future investigations including both sexes may help clarify potential sex‐specific responses to melatonin in smoking cessation.

Limited evidence exists regarding the effects of melatonin on smoking‐related outcomes in female or mixed populations. However, prior studies suggest that sex‐related differences in circadian rhythm and neuroendocrine responses may influence treatment outcomes. Therefore, such considerations further support the need for studies including both sexes to improve external validity. The current findings should be interpreted with caution, as no causal inference can be established. In addition, the exclusive inclusion of male participants introduces a potential gender bias, which may further limit the applicability of the results to female COPD populations.

## 5. Conclusion

Melatonin supplementation was associated with a transient improvement in sleep quality, without demonstrating significant or clinically meaningful benefits in smoking reduction, nicotine dependence, craving, or psychological outcomes compared to placebo. These findings suggest that while melatonin may be a useful adjunct for improving sleep during smoking cessation efforts, it is unlikely to influence the core behavioral or dependence‐related aspects of smoking. Due to the limited sample size and study design constraints, no causal inference can be definitively established from the present findings, and the results should be interpreted with caution. Future research with larger, adequately powered samples, varied dosing regimens, or integration with targeted behavioral or pharmacological cessation interventions may clarify the potential adjunctive role of melatonin in smoking cessation treatment.

## Author Contributions

F.S. and M.G.J.: conception and design of the study; N.H., S.M., and F.H.: collected the data; N.H. and V.R.: prepared the placebo tablets, F.S. and M.M.: worked on the statistical analysis. All authors helped with the preparation of the manuscript.

## Funding

The manuscript was financially supported by a grant from the Research and Technology Department of the Shahid Sadoughi University of Medical Sciences (grant number: 15171), Yazd, Iran. The sponsor took part in the design of the study and approved the final version of the manuscript.

## Disclosure

All authors read and approved the final manuscript.

## Ethics Statement

All stages of the study were approved by the ethics committee of the Shahid Sadoughi University of Medical Sciences of Yazd (Ethic ID: IR.SSU.MEDICINE.REC.1402.137). According to the research ethics guide, all patient information was completely confidential and all stages of the research were performed with patient satisfaction. Written informed consent was obtained for every participant before administration of any study intervention. The participants did not receive a monetary stipend.

## Consent

Please see Ethics Statement.

## Conflicts of Interest

The authors declare no conflicts of interest.

## Data Availability

All data generated or analyzed during this study are included in this published article.
